# Exploring the Chemical Profiles and Biological Values of Two *Spondias* Species (*S. dulcis* and *S. mombin*): Valuable Sources of Bioactive Natural Products

**DOI:** 10.3390/antiox10111771

**Published:** 2021-11-05

**Authors:** Kouadio Ibrahime Sinan, Gokhan Zengin, Dimitrina Zheleva-Dimitrova, Reneta Gevrenova, Marie Carene Nancy Picot-Allain, Stefano Dall’Acqua, Tapan Behl, Bey Hing Goh, Patrick Tang Siah Ying, Mohamad Fawzi Mahomoodally

**Affiliations:** 1 Biochemistry and Physiology Research Laboratory, Department of Biology, Science Faculty, Selcuk University, Konya 42130, Turkey; sinankouadio@gmail.com; 2 Department of Pharmacognosy, Faculty of Pharmacy, Medical University—Sofia, 2 Dunav Str., 1000 Sofia, Bulgaria; dzheleva@pharmfac.mu-sofia.bg (D.Z.-D.); rgevrenova@pharmfac.mu-sofia.bg (R.G.); 3 Department of Health Sciences, Faculty of Medicine and Health Sciences, University of Mauritius, Réduit 80837, Mauritius; picotcarene@yahoo.com; 4 Department of Pharmaceutical and Pharmacological Sciences, University of Padova, Via Marzolo 5, 35131 Padova, Italy; 5 Chitkara College of Pharmacy, Chitkara University, Punjab 140401, India; tapanbehl31@gmail.com; 6 Biofunctional Molecule Exploratory (BMEX) Research Group, School of Pharmacy, Monash University Malaysia, Bandar Sunway 47500, Malaysia; goh.bey.hing@monash.edu; 7 College of Pharmaceutical Sciences, Zhejiang University, Hangzhou 310058, China; 8 Chemical Engineering Discipline, School of Engineering, Monash University, Selangor 47500, Malaysia; patrick.tang@monash.edu

**Keywords:** *Spondias*, enzyme inhibition, gallic acid, natural antioxidants, bioactive agents

## Abstract

*Spondias* species have been used in traditional medicine for different human ailments. In this study, the effect of different solvents (ethyl acetate, methanol, and water) and extraction methods (infusion, maceration, and Soxhlet extraction) on the enzyme inhibitory activity against acetylcholinesterase, butyrylcholinesterase, tyrosinase, α-amylase, α-glucosidase, and antioxidant properties of *S. mombin* and *S. dulcis* leaves and stem bark were evaluated. Ultra-high-performance liquid chromatography–high resolution mass spectrometry (UHPLC-HRMS) yield in the identification and/or annotation of 98 compounds showing that the main secondary metabolites of the plant are gallic and ellagic acids and their derivatives, ellagitannins, hydroxybenzoic, hydroxycinnamic, acylquinic acids and flavonols, flavanones, and flavanonols. The leaves infusion of both *Spondias* species showed highest inhibition against acetylcholinesterase (AChE) (10.10 and 10.45 mg galantamine equivalent (GALAE)/g, for *S. dulcis* and *S. mombin*, respectively). The ethyl acetate extracts of the stem bark of *S. mombin* and *S. dulcis* actively inhibited α-glucosidase. Methanolic extracts of the leaves and stem bark exhibited highest tyrosinase inhibitory action. Antioxidant activity and higher levels of phenolics were observed for the methanolic extracts of *Spondias.* The results suggested that the *Spondias* species could be considered as natural phyto-therapeutic agents in medicinal and cosmeceutical applications.

## 1. Introduction

The use of plants as sources of drugs and bioactive compounds have been attracting considerable scientific attention over the past decades, considering not only the well-known medicinal species but also plants used in traditional medicines of specific countries and part of the world and different edible plants. In particular the study and use of traditionally used plants has been fueled by the recognition of the importance of medicinal plants in the health care system as well as the increased public interest for the use of natural products. In the quest for novel beneficial compounds from plants, ethnobotanical documentation can provide the basis for pharmacological studies. Furthermore, the study of food plants can be of great interest because they contain many classes of bioactive constituents, as polyphenols terpenes, limonoids, carotenoids, each presenting significant biological activities [1], furthermore exploration of food plants may be attractive due to the expected lower toxicity compared to some medicinal species.

For the past decade, the enzyme inhibition has been one of the most popular topics in medical and pharmaceutical applications. In these applications, several enzymes, have been selected as targets that play a key role in the pathologies of some diseases, including Alzheimer’s disease, diabetes mellitus, or obesity. For example, inhibiting acetylcholinesterase increases acetylcholine levels in the synaptic cleavage, so this fact could help to enhance cognitive functions in Alzheimer’s disease [2]. In diabetes mellitus patients, controlling blood glucose levels after a high-carbohydrate diet is the most important treatment strategy. In this sense, the inhibition of amylase and glucosidase is a cornerstone of the strategy and this mechanism could control postprandial glucose level after the diet [3]. Tyrosinase is a key enzyme in the synthesis of melanin and thus inhibiting tyrosinase may help to skin disorders and hyperpigmentation problems [4]. Taken together, the discovery and development of new and safer enzyme inhibitors can help treat the above diseases.

*Spondias* genus, belonging to the Anacardiaceae family, comprises of 18 species distributed in tropical, subtropical, and temperate regions of the globe [5]. Sameh et al. (2018) published a comprehensive review reporting the multiple biological activities of different *Spondias* species [6]. Some species are known for their edible fruits, for example Yellow mombin (*Spondias mombin* L.) a tropical fruit that present high levels of potassium, magnesium, phosphorus, and copper, as well as rich in polyphenols and carotenoids especially Zeinoxanthin and β-criptoxanthin [7]. As a member of the *Spondias* genus, *S. mombin* is a deciduous erected tree that grows up to 15–20 m with a trunk measuring 60–75 cm wide. The plant is commonly found in tropical America and has also been naturalized in parts of Africa and Asia [8]. The leaves and young leaves of *S. mombin* are used in traditional cuisine. The young leaves are cooked as greens and the green fruits pickled in vinegar while ripe fruits and are used to make wine [9].

In ethnomedicine, decoction of *S. mombin* crushed leaves with lemon is used as anthelminthic and against gallbladder stones; an infusion made from *S. mombin* flowers and leaves is used to relieve stomach ache, biliousness, cystitis, urethritis, eye, and throat inflammations; a decoction of the young leaves is used against diarrhea and dysentery by Belizeans [10]. *S. mombin* has been used to manage prostatitis and herpes labialis, intestinal disorders, diabetes, typhoid fever, and as an abortifacient [9,11]. Recent studies conducted on zebrafish showed that the hydroethanolic extract of *S. mombin* leaves was associated with the anxiolytic and antidepressant activities [12]. *S. mombin* leaves have also been reported to possess antioxidant, anti-inflammatory, hepatoprotective, sedative, antiepileptic, antipsychotic, anti-α-amylase, and antileishmanial activity effects and was found to ameliorate gastric ulceration via oxidative and proton pump inhibition [8,11,13,14].

*S. dulcis* (*S. cytherea)* produce fruits that are called Otaheite apple or Golden apple [15], but also known as ambarella, cajarana, and cassemango. The ripe fruit can be used for juices and jam beverages; the fruit is also consumed raw. Young leaves are used as seasoning or cooked as a vegetable while the mature leaves are used in salads. Cambodian folk populations have been using *S. dulcis* bark against diarrhea; the fruits of *S. dulcis* were used against itchiness, internal ulceration, sore throat, skin inflammation, to enhance sight, and treat eye infections [16,17]. Shawkat et al. (2013) previously reported the antimicrobial, antioxidant, and thrombolytic activity of *S. dulcis* fruits and leaves [17].

Although, *S. dulcis* and *S. mombin* are widely spread and have been extensively used in both as food and as traditional medicine for the management of multiple ailments, limited data have been reported on their biological properties and the chemical characterization of the *Spondias* species [18,19]. This study was therefore designed to focus on the comparative assessment of the leaves and stem barks extracts obtained with different solvents (water, ethyl acetate, and methanol) and different techniques (infusion, maceration, and Soxhlet extraction**)** from *S. dulcis* and *S. mombin* with regard to biological properties (antioxidant and enzyme inhibitory effects) and chemical characterization.

## 2. Materials and Methods

### 2.1. Plant Material and Preparation of Extracts

The *Spondias* species were obtained in a field study in Côte d’Ivoire (Gbêkê region, Brobo city, Prikro village) during summer 2019. The plant samples were authenticated by botanist Dr. Kouadio Bene. The plant samples were deposited at the Selcuk University, Department of Biology, Kenya. Stem barks and leaves were carefully separated, and they were dried in an air ventilated condition for 10 days without sunshine. For extracts preparation, infusion, Soxhlet (SOX), and maceration (MAC) were used. The details for the performed extractions are given in the supplemental material. Methanol and ethyl acetate were removed by using rotary evaporator, while infusion was lyophilized. All dried extracts were stored at +4 °C until further uses.

### 2.2. Chemicals and Reagents

Gallic (3), ellagic (38), protocatechuic (45), neochlorogenic (48), caffeic (49), chlorogenic (50), syringic acids (53), myricitrin (59), isoquercitrin (60), hyperoside (64), kaempferol 3-*O*-glucoside (67), quercitrin (68), isorhamnetin 3-*O*-glucoside (72), quercetin (77), myricetin (78), kaempferol (80), (+)-catechin (82), and prunin (87) were obtained from Extrasynthese (Genay, France). All reagents and solvents were of analytical grade.

### 2.3. Profile of Bioactive Compounds

Total phenolic content (TPC), total flavonoid content (TFC), total flavanol (TFvL), and total phenolic acid (TPaC) contents of *Spondia* extracts were determined as previously described [20,21]. The results were expressed as equivalent of gallic acid (GAE) for TPC, rutin (RE) for TFC, caffeic acid (CAE) for TpaC, and catechin (CE) for TFvL.

### 2.4. Chromatographic Separation

Separation was achieved on an UHPLC system Dionex Ultimate 3000RSLC (ThermoFisher Scientific, Inc., Waltham, MA, USA) with reversed phase column Kromasil Eternity XT C18 (1.8 µm, 2.1 × 100 mm) column maintained at 40 °C. The binary mobile phase consisted of A: 0.1% formic acid in water and B: 0.1% formic acid in acetonitrile. The run time was 33 min. The following gradient was utilized: the mobile phase was held at 5% B for 1 min, gradually turned to 30% B over 19 min, increased gradually to 50% B over 5 min, increased gradually to 70% B over 5 min, and finally increased gradually to 95% over 3 min. The system was then turned to the initial condition of 5% B and equilibrated over 4 min. The flow rate and the injection volume were set to 300 µL/min and 1 µL, respectively. The effluents were connected on-line with a Q Exactive Plus Orbitrap mass spectrometer where the compounds were detected [22].

### 2.5. Mass Chromatography Conditions

Mass analyses were carried out on a Q Exactive Plus mass spectrometer (ThermoFisher Scientific, Inc.) equipped with a heated electrospray ionization (HESI-II) probe (ThermoScientific). The tune parameters were as follows: spray voltage 3.5 kV; sheath gas flow rate 38; auxiliary gas flow rate 12; spare gas flow rate 0; capillary temperature 320 °C; probe heater temperature 320 °C; and S-lens RF level 50. Acquisition was acquired at Full-scan MS and Data Dependent-MS2 modes. Full-scan spectra over the m/z range 100 to 1500 were acquired in negative ionization mode at a resolution of 70,000. Other instrument parameters for Full MS mode were set as follows: AGC target 3e6, maximum ion time 100 ms, number of scan ranges 1. For DD-MS2 mode, instrument parameters were as follows: microscans 1, resolution 17,500, AGC target 1e5, maximum ion time 50 ms, MSX count 1, isolation window 2.0 m/z, stepped collision energy (NCE) 20, 40, and 70 eV. Data acquisition and processing were carried out with Xcalibur 4.2 software (ThermoScientific) [22].

### 2.6. Determination of Antioxidant and Enzyme Inhibitory Effects

The antioxidant activity of *Spondias* samples was determined by 1,1-diphenyl-2-picryhydrazyl (DPPH) and 2,2′-azino-bis(3-ethylbenzothiazoline-6-sulfonic acid) (ABTS) radical scavenging, cupric reducing antioxidant capacity (CUPRAC), ferric reducing antioxidant power (FRAP), metal chelating (MCA), and phosphomolybdenum (PBD) assays [23,24]. DPPH, ABTS, CUPRAC, and FRAP activities were expressed as mg trolox equivalents (TE)/g extract. In MCA, the data were presented as mg EDTA equivalents (EDTAE)/g extract, whereas in PBD assay, the results were provided as mmol TE/g extract. Acetylcholinesterase (AChE) and butyrylcholinesterase (BChE) inhibition was assessed as described in Uysal et al. [23] and Grochowski et al. [24] and, expressed as mg galantamine equivalents (GALAE)/g extract, while amylase and glucosidase inhibition [23,24] was reported as mg acarbose equivalents (ACAE)/g extract. Tyrosinase inhibition was also expressed as kojic acid equivalents (KAE)/g extract [23,24].

### 2.7. Statistical Analysis

Statistical analyses were performed under Xlstat v 2020 and R v 3.6.2 software, respectively. Outcomes were done as mean ± standard deviation and statistical difference was determined by one-way analysis of variance (ANOVA). At *p* < 0.05 level, Turkey’s post hoc test was done for the sample’s comparisons. Then, both total chemical contents and bioactivities datasets were scaled, centered, and submitted to the principal component analysis (PCA). Subsequently, Cluster Image Map (CIM) analysis was performed on the result of PCA. Similarly, Cluster Image Map (CIM) analysis was done on the relative content of identified chemical compounds by using the peak area data matrix derived from UHPLC-MS analysis. Before analysis, the data matrix was logarithmic transformed. Both Cluster Image Map analysis were based on “Ward” and “Euclidean” as linkage rule and distance, respectively.

## 3. Results and Discussion

Based on the analytical parameters (retention times, MS and MS/MS accurate masses, fragmentation patterns and comparison with reference standards and literature data), 98 metabolites including gallic and ellagic acids and derivatives, ellagitannins, hydroxybenzoic, hydroxycinnamic, acylquinic acids and derivatives, flavonols, flavanones, flavanonols, flavan-3-ols, and others are unambiguously identified. A list of compounds is reported in Table 1. The fragmentation of the identified metabolites is given in supplemental material (Table S1).

### 3.1. Gallic Acid and Galloyl Derivatives

**Figure 1:**
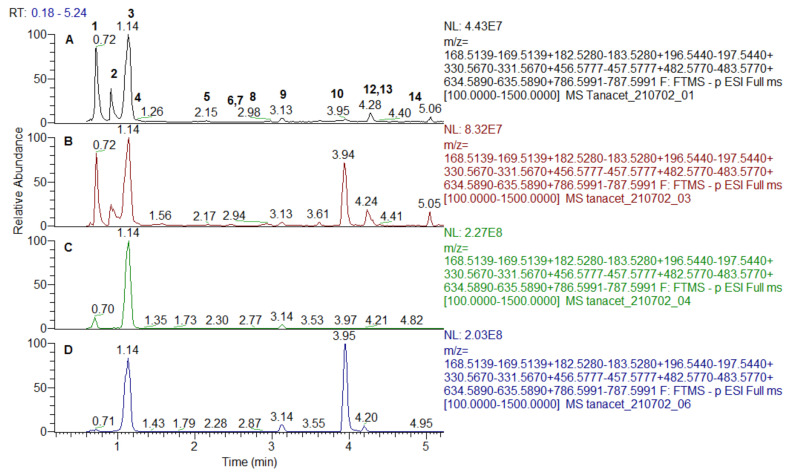
Extracted ion chromatogram of gallic acid and galloyl derivatives from *Spondias dulcis* extracts: (**A**)—leaves water extracts; (**B**)—leaves methanol extract; (**C**)—stem bark water extract; (**D**)—stem bark methanol extract.

**Figure 2:**
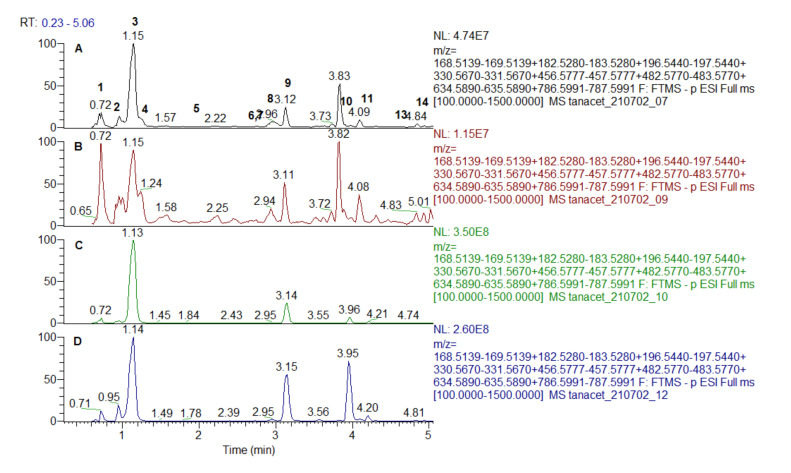
Extracted ion chromatogram of gallic acid and galloyl derivatives from *Spondias mombrin* extracts: (**A**)—leaves water extracts; (**B**)—leaves methanol extract; (**C**)—stem bark water extract; (**D**)—stem bark methanol extract.

Extracted ion chromatogram of the galloyl derivatives are depicted in Figure 1 and Figure 2. MS/MS fragmentation pathway of gallic acid (GA) (3) and galloyl derivatives yielded characteristic ions at *m/z* 169.013 [GA-H]^−^, 151.003 [GA-H-H_2_O]^−^, 125.023 [GA-H-CO_2_]^−^, and 107.012 [GA-H-H_2_O-CO_2_]^−^ (Table 1).

MS/MS spectra of three isobaric compounds (1, 2, and 4) with [M-H]^−^ at *m/z* 331.067 were acquired (Table 1). Compounds 1 and 2 afforded fragment ions resulting from hexose cross cleavages based on the loss of –CHOH: ^0,4^Hex (−60 Da) at *m/z* 271.046, ^0,3^Hex (−90 Da) at *m/z* 241.035 and ^0,2^Hex (−120 Da) at *m/z* 211.024. Similar to the MS/MS pattern of caffeoyl hexoses, they were ascribed as isomeric galloyl-hexoses (sugar esters), while 4 was tentatively identified as gallic acid-*O*-hexoside [22]. In the same manner, 5 and 8 were assigned to digalloyl hexoside and digalloyl hexose. Regarding 11 and 13, three galloyl residues were evidenced by the transitions 635.089 → 483.079 [M-H-galloyl]^−^, 483.079 → 313.057 [M-H-galloyl-H_2_O]^−^, and 313.057→169.013 [GA-H]^−^ (Table 1). Tetragalloyl hexoside (14) was witnessed by the subsequent losses of galloyl residues at *m/z* 465.065 [M-H-2galloyl-H_2_O]^−^ and 295.046 [M-H-3galloyl-2H_2_O]^−^. Herein, we report for the first time galloyl-, digalloyl-, and trigalloyl hexosides in both studied species.

Compounds 6 and 7 shared the same [M-H]^−^ at *m/z* 495.078 (Table 1). They were tentatively assigned to digalloylquinic acids as indicated by the common abundant MS/MS ion at *m/z* 343.067 derived from the loss of a galloyl residue and the prominent ions at *m/z* 191.055 (deprotonated quinic acid) and “dehydrated” quinic acid at *m/z* 173.045 [25].

Methylgallate (9) and dimetylgallate (12) were deduced from the typical loss of methyl radical (•CH_3_) (−15 Da) at *m/z* 168.005 (9) and consequent losses of methyl radicals at *m/z* 182.0211 and 166.998 (12) (Table 1). In (-) ESI-MS/MS spectrum 10 ([M-H]^−^ at *m/z* 457.078) yielded fragment ions at *m/z* 305.067 [M-H-galloyl]^−^, 287.056 [M-H-GA]^−^, and 169.013 [GA-H]^−^ (a base peak) indicating a galloyl derivative. On the other hand, epigallocatechin was observed at *m/z* 287.056 (C_15_H_21_O_6_) together with a series of fragment ions at *m/z* 269.045 [M-H-GA-H_2_O]^−^, 261.076 [M-H-GA-CO]^−^, 243.066 [M-H-GA-CO_2_]^−^, and 219.066 [M-H-GA-CO-CO_2_]^−^. Thus, 10 was tentatively identified as epigallocatechin gallate previously reported in *S. tuberosa* [26].

### 3.2. Ellagic Acid Derivatives and Ellagitannins

**Figure 3:**
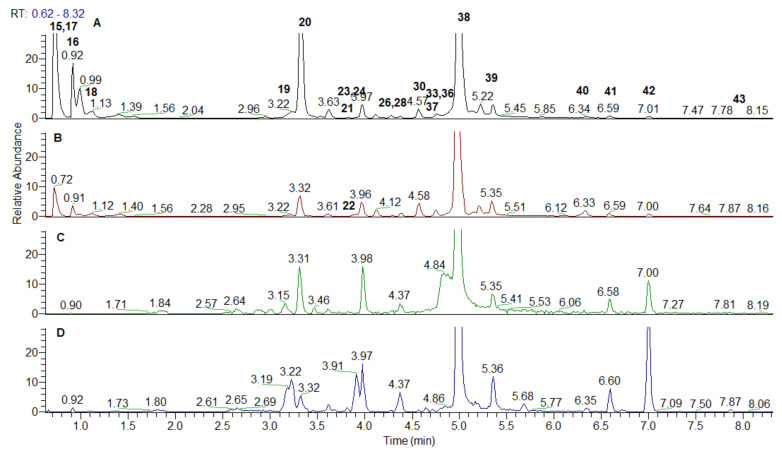
Extracted ion chromatogram of ellagitannins and ellagic acid derivatives from *Spondias dulcis* extracts: (**A**)—leaves water extracts; (**B**)—leaves methanol extract; (**C**)—stem bark water extract; (**D**)—stem bark methanol extract.

**Figure 4:**
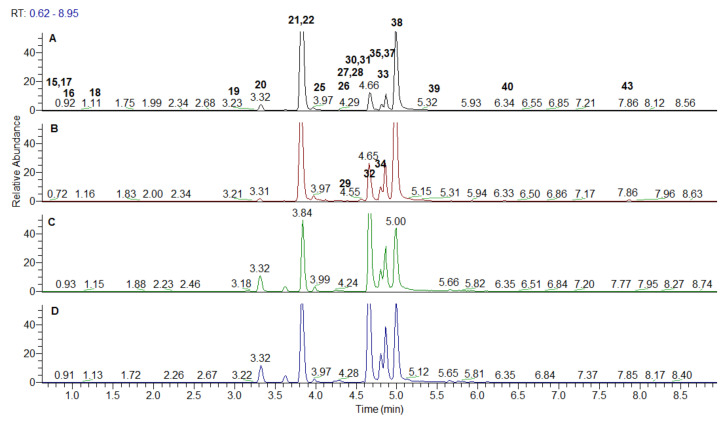
Extracted ion chromatogram of ellagitannins and ellagic acid derivatives from *Spondias mombrin* extracts: (**A**)—leaves water extracts; (**B**)—leaves methanol extract; (**C**)—stem bark water extract; (**D**)—stem bark methanol extract.

*Spondias* ellagitannins include monomeric HHDP (hexahydroxydiphenoyl) unit-bearing ellagitannins (15–19, 21, 24, 27, and 35) and dimeric derivatives 31 and 36 (Table 1, Figure 3 and Figure 4). All of them could be referred to Okuda’s type II ellagitannins [27], while 22 and 29 possesses dehydrohexahydroxydiphenoyl (DHHDP) unit, being Okuda’s type III tannin (dehydroellagotannin) [28]. Main criteria in the peak annotation of the ellagitannins were the neutral mass losses of 152 Da (galloyl moiety), 170 Da (gallic acid), 302 Da (HHDP), 332 Da (galloyl hexose), and 482 Da (HHDP hexose) as was reported in the literature [29,30]. Afterwards, the fragmentation patterns yielded the diagnostic fragment ion at *m/z* 300.999 resulting from the spontaneous lactonization of HHDP into ellagic acid (EA) and *m/z* 169.013 consistent with a galloyl residue [29]. MS/MS fragmentation pathway of EA (38) afforded characteristic ions corresponding of the neutral losses of CO and CO_2_ at *m/z* 257.010 [EA-H-CO_2_]^−^, 245.009 [EA-H-2CO]^−^, 217.013 [EA-H-3CO]^−^_,_ and concomitant losses at *m/z* 229.013 [EA-H-CO-CO_2_]^−^, 201.019 [EA-H-2CO-CO_2_]^−^, 185.023 [EA-H-2CO-2CO_2_]^−^, 173.023 [EA-H-3CO-CO_2_]^−^, 145.028 [EA-H-4CO-CO_2_]^−^, 129.033 [EA-H-3CO-2CO_2_]^−^, and 117.033 [EA-H-5CO-CO_2_]^−^ (Table 1). Compound 38 was unambiguously identified by comparison with reference standard.

MS/MS spectra of the isobaric pair 15 and 16 with [M-H]^−^at *m/z* 481.062 were acquired in both *Spondias* species (Table 1). The typical fragment ion at *m/z* 300.999 resulted from the loss of 180 Da (Hex + H_2_O) indicating HHDP-hexoside.

Four isobars 17–19 and 21 shared the same [M-H]^−^at *m/z* 633.073; the abundant fragment ion at *m/z* 300.999 suggested the loss of 332 Da and galloyl-hexose moiety (Table 1). Accordingly, the aforementioned compounds were ascribed as galloyl-HHDP-hexoside isomers. This assumption was in line with the presence of monomeric ellagitannin corilagin previously annotated in *S. mombrin* and *S. tuberosa* [26]. In the same way, digalloyl-HHDP-hexoside isomers 24, 27 and 35 at *m/z* 785.084 [M-H]^−^ were evidenced at by the characteristic losses of 484 Da (152 + 152 + 162 + 18) consistent with two galloyl and hexosyl residues. Di-HHDP-hexoside (36) ([M-H]^−^ at *m/z* 783.075) was found in *S. dulcis* leaves witnessed by the loss of HHDP-hexose (482 Da) at *m/z* 300.999. Compound 31 from *S. mombrin* leaves was deduced from the transitions 953.090 → 463.168 ([M-H-HHDP-H_2_O-GA]^−^ and 463.168 → 300.999 ([EA-H]^−^] (Table 1). It was assigned to galloyl-bisHHDP-hexoside [26]. Type III ellagitannin galloyl-HHDP-DHHDP-hexoside (22) was evidenced by the fragment ions at *m/z* 463.052 [M-H-C_21_H_12_O_14_]^−^, 343.009 and 300.999 suggesting the concomitant loss of DHHDP and gallic acid (−488 Da), subsequent cross-ring cleavage of hexose unit (^0,2^X, −120 Da) and EA, respectively. Compound 22 was ascribed to geraniin, while additional galloyl residue was found in 29 (galloyl-geraniin) (Table 1). Both compounds **22** and **29** were previously isolated from *S. mombrin* leaves and stems [31]. Based on the prominent ions at *m/z* 315.011 [M-H-Hex]^−^ and *m/z* 299.9905 [M-H-Hex-CH_3_]^−^, compounds **28** and **37** were assigned as methylellagic acid-*O*-hexoside isomers (Table 1). In the same manner, the tentative structure of dimethylellagic acid-hexoside was proposed for the isobaric compounds **39** and **41**.

Methylellagic (40) and dimetylallagic acid (43) were annotated in both species, while trimethylellagic acid (42) was evidenced only in *dulcis* leaves. In addition, EA-deohyhexoside (33) was commonly found in the studied extracts, while EA-hexoside/pentoside (25, 32, 33) were evidenced only in the *S. mombrin* leaves methanol extract.

Compound 20 ([M-H]^−^ at *m/z* 291.045, C_13_H_7_O_8_, 0.755 ppm) gave and a base peak at *m/z* 247.025 and a series of low abundant ions at 219.030 [M-H-CO_2_-CO]^−^, 191.034 [M-H-CO_2_-2CO]^−^, 163.039 [M-H-CO_2_-3CO]^−^, 135.044 [M-H-CO_2_-4CO]^−^, and 107.049 [M-H-CO_2_-5CO]^−^ (Table 1). Based on the comparison with date from the literature, 20 was annotated as brevifolin carboxylic acid [26,30]. Concerning 26 ([M-H]^−^ at *m/z* 247.025), there was mass difference of 44 Da with 20 indicating that the associated molecule missed a carboxyl group; the MS/MS spectrum matched that of 20 (Table 1). Accordingly, 26 was annotated as brevifolin. Compound 30 ([M-H]^−^ at *m/z* 305.031) afforded a prominent ion at *m/z* 273.004 ([M-H-CH_3_OH]^−^ (a base peak) suggesting a methyl ester of 20—methylbrevifolin carboxylate.

### 3.3. Hydroxybenzoic, Hydroxycinnamic, Acylquinic Acids and Derivatives

Based on the retention times, accurate masses, MS/MS fragmentation patterns, and comparison with reference standards and literature data, six hydroxybenzoic (44–46, 51, 53, and 54), two hydroxycinnamic (47 and 49), and three acylquinic (48, 50, and 52) acids and derivatives were unambiguously identified (Table 1) [22].

### 3.4. Flavonols

The flavonol aglycones quercetin (77), myricetin (78), and kaempferol (80) were identified by comparison with the retention times in UHPLC-HRMS and MS/MS fragmentation fingerprints of reference standards (Table 1).

In general, MS/MS fragmentation pathways of flavonoid glycosides demonstrated neutral mass losses of 162.053, 146.058, 132.042, and 176.033 Da consistent with hexose, deoxyhexose, pentose, and hexuronic acid, supported by the Retro-Diels-Alder (RDA) cleavages of the flavonoid skeleton [22]. Compounds 55, 56, 60, 63, 64, 65, 68, and 70 showed a product ion at *m/z* 301.035, together with RDA ions at *m/z* 151.002 [^1,3^A]^−^, 121.027 [^1,2^B]^−^, and 107.012 [^0,4^A]^−^, suggesting the presence of the quercetin core [32]. The 3-glycosylation position of 60, 64, and 68 was determined by the low abundant Y_0_^-^ ion (*m/z* 301.035) compared with the radical aglycone ion [Y_0_-H]^−^ (*m/z* 300.027), as typical MS/MS fragmentation behavior of flavonoid 3-*O*-glycosides, instead of 7-*O*-glycosides [32]. Accordingly, and based on the comparison to the reference standards, 60, 64, and 68 were identified as isoquercitrin, hyperoside, and quercitrin. Regarding 63 and 70, a base peak of the radical aglycone ion at *m/z* 300.027, allowed for the annotation of quercetin 4′-*O*-glycosilation. Based on the neutral losses of 176.033 and 162.053, 63 and 70 could be related to quercetin 4′-*O*-hexuronide and quercetin 4′-*O*-hexoside, respectively (Table 1). Compounds 55 [M-H]^−^ at *m/z* 625.141 and 56 [M-H]^−^ at *m/z* 595.130 corresponded to dihexoside and hexoside-pentoside of quercetin, respectively. The absence of interglycosidic bonds cleavages revealed a 3-*O*-position of diglycoside chains [33]. In the same manner, due to the fragment ions at *m/z* 285.039, 284.032, 151.003, and 107.012, compounds 62, 67, 69, 73, and 75 were ascribed as kaempferol-*O*-glycosides. Diagnostic ions at *m/z* 317.029 [Y_0_]^−^ and 316.022 [Y_0_-H]^−^, supported by RDA ions at *m/z* 137.023 [^1,2^B]^−^, 164.010 [^1,3^B-H]^−^, and 178.997 [^1,2^A]^−^ could associate 58, 59, 61, 71, and 74 to myricetin derivatives (Table 1). Regarding 66 with [M-H]^−^ at *m/z* 477.103, consequent losses of deoxyhexose and one •CH_3_ at *m/z* 331.046, and 316.022 suggested methylmyricetin-*O*-deoxyhexoside. The methoxyl group could be in ring B, deduced from the ion at *m/z* 136.014 (^1,2^B-•CH_3_). Similar to the aforementioned flavonol, 72 possessed a methoxyl group in a ring B deduced from the prominent ion at *m/z* 300.027 arising from loss of methyl radical from the deprotonated aglycon ion [M-H-Glc-•CH_3_]^−^ and fragments at *m/z* 151.002 and 107.012 assigned to A-ring (Table 1). Thus, 72 was identified as isorhamnetin 3-*O*-glucoside confirmed by a comparison with reference standard. In contrast to 72, peaks 76 and 79 produced A-ring fragments at *m*/*z* 165.018 [^1,3^A]^−^ and 121.028 [^0,4^A]^−^, retaining the methoxyl group in ring A. Accordingly, 76 and 79 were related to rhamnetin 3-*O*-hexoside and rhamnetin *O*-deoxyhexoside [34] (Table 1).

### 3.5. Flavanones, Flavanonols and Flavan 3-ols

Compound 82, [M-H]^−^ at *m/z* 289.071 gave a fragment ions at *m/z* 271.060 [M-H-H_2_O]^−^, 245.081 [M-H-CO_2_]^−^, 205.050, 203.070 (cleavage of the A-ring of flavan-3-ol), 179.033 (cleavage of B-ring), and RDA ions at *m/z* 151.038 [^1,3^B]^−^, 137.022 [^1,3^A]^−^, and 109.028 [^0,4^A + 2H]^−^ [35]. Based on the comparison to a reference standard, 72 was unambiguously identified as (+)-epicatechin. Compounds 81, 88, and 90 demonstrated similar fragmentation patterns. Compound 81 differs from 72 by one hexose (165.053 Da) and was assigned as (epi)catechin-*O*-hexoside. Compounds 88 and 90 revealed a fragment ion at *m/z* 169.013, corresponding to a galloyl residue and were tentatively ascribed to (epi)catechin-gallate (Table 1).

Three isobars 83, 84, and 86 shared the same deprotonated molecular ion at *m/z* 465.103. MS/MS spectra of compounds produced fragment ion at *m/z* 304.054 [M-H-Hex], corresponding to taxifolin *O*-hexosides. Prominent fragment ions at *m/z* 259.061 [Agl-H-CO_2_]^−^, 241.050 [Agl-H-CO_2_-H_2_O], and RDA ions at *m/z* 178.997 [^1,2^A]^−^, 151.002 [^1,3^A]^−^, 149.023 [^1.3^B]^−^, and 125.023 [^1,4^A]^−^ confirmed the presence of taxifolin [36]. Based on the ratio between deprotonated molecular ion and fragments at *m/z* 304.054 [Y_O_^-^] and 303.051 [Y_O_-H]^−^, the position of glycosylation of taxifolin can be distinguished (Table 1). The flavanon aglycon naringenin (93) ([M-H]^−^ at *m/z* 271.061) was witnessed by RDA ions at *m/z* 151.002 [^1,3^A]^−^, 119.048 [^1,3^B]^−^, and 107.012 [^0,4^A]^−^ [33]. Compounds 87 and 91 ([M-H]^−^ at *m/z* 433.114) differed from 93 by 162.05 Da, corresponding to hexosides of naringenin. Compound 87 was unambiguously identified as naringenin 7-*O*-glycoside, deduced from the base peak at *m/z* 271.061 [Agl-H]^−^ and comparison with reference standard. Regarding 91, a series of fragment ions at *m/z* 343.082 [M-H-90]^−^, 283.062 [M-H-150]^−^, and a base peak at *m/z* 313.072 [M-H-120]^−^ were indicative for naringenin 8-*C*-hexoside [37]. In the same manner 94 could be related to flavanon aglycon pinocembrin, and 92 to its *O*-hexoside, while 89 was ascribed to eriodictiol 7-*O*-hexoside [33].

### 3.6. Others

Compound 95 [M-H]- at m/z yielded a precursor ion at *m/z* 341.108 (C_12_H_22_O_11_) together with the fragment ions at *m/z* 179.055 ([M-H-Hex]^−^ and 89.022 ([M-H-Hex-90]^−^ suggesting disaccharide sucrose, previously found in *Spondias* species [26]. Regarding 98, a base peak at *m/z* 151.038, together with prominent fragment ions at *m/z* 136.015 and 121.028 are in accordance with the furofuran lignan pinoresinol [38]. In the MS/MS spectrum of 97, the main fragment ions were observed at *m/z* 341.066 and 217.013, which were similar to those of cinchonain Ib. The ion at *m/z* 341.066 was produced from the neutral loss of C_6_H_6_O_2_ (110 Da), which confirmed the existence of a dihydroxyphenyl group. The ion at *m/z* 217.013 was generated from the neutral losses of C_6_H_6_O_2_ and C_7_H_8_O_2_ (234 Da), produced from the elimination of dihydroxytoluene. Thus, compounds 97 was related to dihydroxylphenylpropanoid-substituted catechin cinchonains Ib [39].

### 3.7. Enzyme Inhibitory Properties

Enzyme inhibition is one of the most attractive topics in medical and pharmaceutical research. For example, the inhibition of acetylcholinesterase is closely related to the alleviation of the observed symptoms of Alzheimer’s disease. As another example, the inhibition of α-amylase and α-glucosidase is the basis of oral anti-diabetic agents for the treatment of diabetes type II. Tyrosinase is also considered to be the main target in the control of skin disorders and hyperpigmentation problems. In this context, we selected the enzymes (cholinesterases, α-amylase, and α-glucosidase and tyrosinase) to determine inhibitory properties of the tested *Spondia* extracts. From Table 2, it was noted that the stem bark maceration-EA of *S. dulcis* and the leaves infusion and Soxhlet-MeOH as well as the stem bark maceration-EA (no stir) of *S. mombin* (10.33, 10.45, 10.37, and 10.31 mg GALAE/g, respectively) showed highest inhibition against acetylcholinesterase. The pathogenesis of Alzheimer’s disease is complex and the mechanisms are poorly understood [40], but scientific evidences have attested of the role of cholinesterase enzymes. Acetylcholinesterase inhibition has been advocated in the management of Alzheimer’s disease, since this enzyme is directly linked to the hydrolysis of the neurotransmitter, acetylcholine, at the synaptic cleft. Moreover, isoquercitrin, previously identified in *S. mombin* hydroethanolic extract [12] and in other *Spondias* species, including *S. tuberosa*, was reported to modulate acetylcholinesterase activity, as well as β- and γ-secretase and Aβ aggregation [41]. Clinical studies have revealed that another cholinesterase enzyme, butyrylcholinesterase, was also implicated in the exacerbation of the health condition of Alzheimer’s disease patients [42,43,44]. These facts support the quest for therapeutic candidates possessing butyrylcholinesterase inhibitory activity. Data presented in Table 2, showed that the different extracts of the leaves and stem bark of the selected *Spondias* species exhibited variable inhibitory action against butyrylcholinesterase, with the highest anti-BChE value exhibited by leaves maceration-MeOH (not stir) of *S. mombin*. However, it was observed that the studied extracts displayed more prominent inhibitory action against acetylcholinesterase compared to butyrylcholinesterase. A previous study carried out by Elufioye et al. (2017) demonstrated the inhibitory action of *S. mombin* leaves methanolic extract on acetylcholinesterase and butyrylcholinesterase, with special focus on the inhibitory action of botulin, campesterol, and phytol, isolated from *S. mombin* leaves [45]. The oxytocic ability of *S. mombin* leaves extracts has been reported, thereby validating its traditional use as abortifacient [45]. In addition, in vivo study has demonstrated that *S. mombin* leaves extract could affect gonadotrophin secretion by the pituitary gland in male Wistar rats [46], advocating the ability of *S. mombin* leaves extract to cross the blood–brain barrier (BBB). The BBB shields the brain from harmful toxins and pathogens, but this protective characteristic is also a hurdle to the entry of therapeutic agents. One of the main reasons why around 98% of newly developed drug candidates fail clinical trial is their inability/poor ability to cross the BBB [47]. Therefore, assessing the potential of new compounds to cross the BBB is crucial. On the other hand, as far as our literature search could establish, this study can be regarded as the first attempt to report the in vitro cholinesterase inhibitory activity of *S. dulcis* leaves and stem bark.

An increasing number of scientific reports describe the relationship between Alzheimer’s disease and diabetes type II. In fact, substantial epidemiological evidence suggest that Alzheimer’s disease and diabetes type II are intertwined conditions [48,49,50]. Kandimalla and colleagues [51] coined the term “type 3 diabetes” to emphasize on the shared molecular and cellular features related to Alzheimer’s disease and diabetes type II. *S. mombin* has been used in traditional medicine to manage diabetes and was also previously reported to inhibit α-amylase [9]. Nevertheless, it is worth mentioning that novel therapeutic strategies for the management of diabetes type II encompass lower inhibition of α-amylase and stronger α-glucosidase inhibition. It has been postulated that the simultaneous inhibition of these carbohydrate hydrolyzing enzymes would result in abnormal bacterial fermentation of undigested carbohydrates in the colon, causing abdominal distention, flatulence, meteorism, and possibly diarrhea [52]. Results gathered from this study showed that both *Spondias* species were poor inhibitors of α-amylase (Table 2). On the other hand, several extracts of *S. mombin* and *S. dulcis* leaves and stem bark actively inhibited α-glucosidase. In contrast no activity was recorded for 11 extracts. *S. dulcis* fruit ethanol extract was reported to inhibit α-glucosidase activity [53].

The ability of the *Spondias* extracts to inhibit tyrosinase activity was assessed and presented in Table 2. In general, methanolic extracts of the leaves and stem bark of the selected *Spondias* species exhibited highest tyrosinase inhibitory action. However, the strongest anti-tyrosinase activity was recorded by stem bark maceration-MeOH (no stir) extract of *S. mombin*. The quest for new tyrosinase inhibitors has mainly been driven by the side effects, namely, contact dermatitis, which is accompanied by rashes, irritation, itchiness, inflamed skin, and pain, caused by currently used depigmenting agents, such as kojic acid [54]. Moreover, the increased public demand for naturally derived products has also been a major factor boosting the search for novel agents. Oyasowo et al. (2018), recently reported the inhibitory action of *S. mombin* methanolic root bark extract against tyrosinase [55]. The application of *S. mombin* in the formulation of a cosmetic product having depigmenting as well as anti-aging and anti-radical action has also been documented. Data gathered from the present study demonstrated that *S. dulcis* also showed potent tyrosinase inhibitory activity. It is worth highlighting that the extracts obtained by infusion of the leaves showed no activity against tyrosinase.

### 3.8. Antioxidant Properties

The role of oxidative stress in the pathogenesis of diabetes type II, Alzheimer’s disease, and skin hyperpigmentation conditions has been extensively documented [56,57,58]. In the present study, a number of assays were used to provide a comprehensive understanding of the antioxidant potential of *S. mombin* and *S. dulcis* leaves and stem bark extracts. Moreover, since many studies have supported the link between total bioactive compounds profile and the antioxidant potential of herbal extracts, the total phenolic, flavonoid, phenolic acid, and flavonol contents of *Spondias* species extracts was assessed [8,11,17]. The antioxidant along with the hepatoprotective effect of *S. mombin* leaf and stem bark methanolic extract has been previously established in vivo. Another group of researchers reported the antioxidant properties of *S. dulcis* fruits and leaves methanolic extracts. From Table 3, methanolic extracts of the selected *Spondias* species possessed highest level of phenolic compounds. The stem bark extracts of both species possessed higher phenolic content compared to the leaves’ extracts. Moreover, stem bark maceration-MeOH (no stir) of both species as well as and stem bark-MeOH of *S mombin* had stronger TPC (240.24; 244.28, and 245.50 mg GAE/g, respectively). The opposite was noted for the flavonoid content, highest TFC was recorded for leaves extracts. In addition, methanolic extracts of *S. dulcis* leaves (40.71–43.41 mg RE/g) showed higher TFC. In most cases, the phenolic acid content was higher in extracts obtained by infusion, however, the higher amount was achieved by *S. mombin* infusion leaves infusion and methanol stem bark obtained by maceration without stirring. Phenolic acids solubility in water and organic solvents such as ethyl acetate and methanol was evaluated Vilas-Boas and colleagues (2018) who reported that extraction temperature and solvent nature will affect the solubility of phenolic acids differently [59]. The highest flavonol content was recorded from *S. mombin* stem bark ethyl acetate extract. Assessment of the total antioxidant capacity of the extracts revealed that extracts showing highest TPC showed highest antioxidant activity. This finding corroborates with a number of studies which have also reported the positive correlation between total antioxidant capacity and TPC. The radical scavenging, reducing power, and metal chelating properties of *S. mombin* and *S. dulcis* leaves and stem bark extracts were summarized in Table 4. Widely used DPPH and ABTS radical scavenging assays showed that in general the methanolic extracts were the most active radical scavengers. Similar trend was observed for the CUPRAC and FRAP assays. The stem bark extracts showed higher radical scavenging and reducing capacity compared to the leaves’ extracts. *S. mombin* ethyl acetate leaves extract obtained by maceration without stirring showed highest metal chelating potential. Findings gathered on the antioxidant potential of the selected *Spondias* species revealed that these species have potential as antioxidant agent with special emphasis on the higher activity of the stem bark.

### 3.9. Data Mining

**Figure 5:**
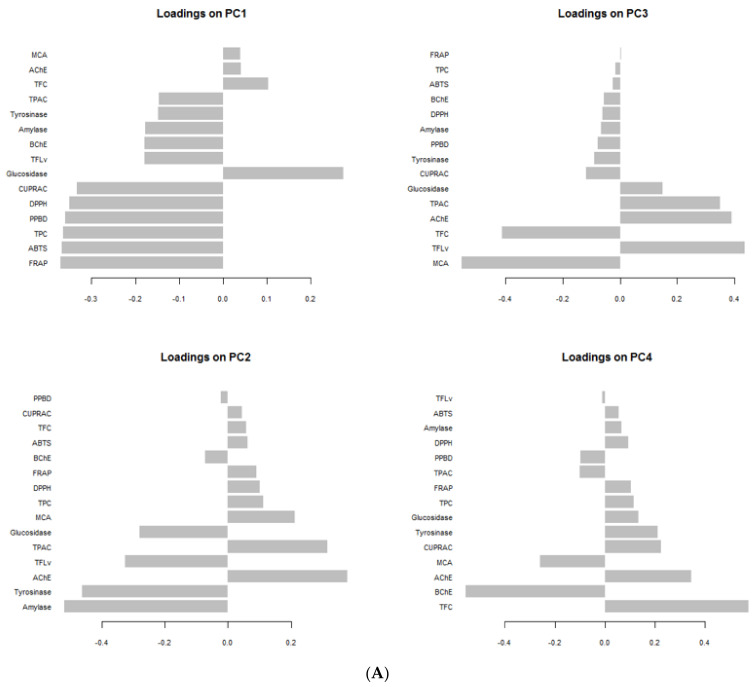

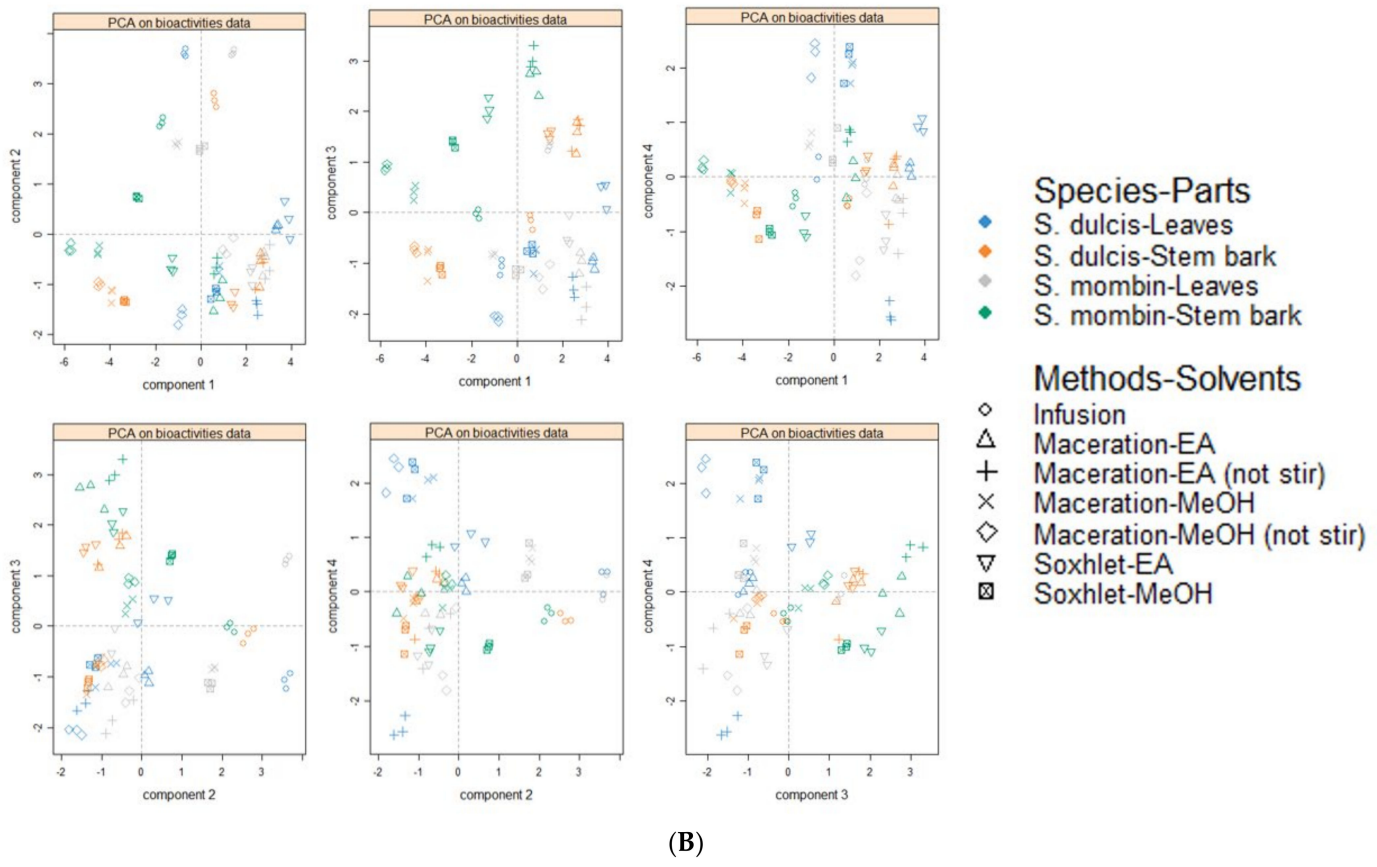
Principal component analysis (PCA) showing the variability of the total chemical composition, antioxidant and anti-enzymatic activity of *Spondias* species. (**A**) Loading plots displaying the relationship between the total chemical composition, antioxidant and anti-enzymatic activity and the four significant PCs. (**B**) Score plots showing the distribution of the samples in the six 2-dimension plans obtained from the four significant PCs.

**Figure 6:**
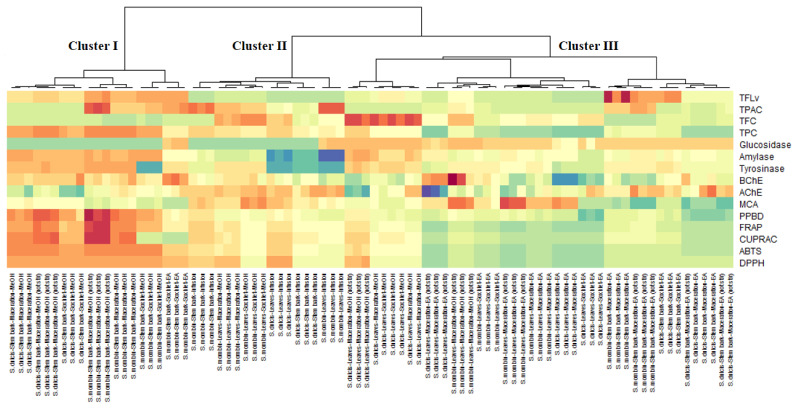
Cluster image map (CIM) analysis of the total chemical composition, antioxidant and anti-enzymatic activity of *Spondias* species (Red color: high concentration or bioactivity, Blue color: low concentration or bioactivity).

Furthermore, using the total chemical compounds and bioactivities datasets, principal component analysis was performed to uncover the variation among the extracts of *Spondias* species. Primarily, the small number of principal component apprehended most of the variation in the data were selected with reference to Kaiser rule [60]. Hence, four dimensions which possessed an eigenvalue higher than 1 and 80% of the variability were retained (Table S2). The bioactivities describing each principal component can be identify by referring to Figure 5A. The first component (PC1) predominantly represented the variation in antioxidant, TPC, and anti-glucosidase, the second component (PC2) largely depicted the variation in anti-amylase, anti-tyrosinase, anti-AChE, TFvL, and TPac, the third component (PC3) referred to variation in MCA, TFvl, and TFC while the fourth component (PC4) was mainly determined by TFC and anti-BChE. Regarding the distribution of the samples on the score plot (Figure 5B) deriving from the four principal components, there was some variability, however, it was not possible to identify clearly the different homogeneous groups. Therefore, a heat-map was created to classify the samples and also to identify the total bioactive compounds and biological activities characterizing each obtained clusters. As shown in Figure 6 three major clusters was obtained. Overall, the samples being in cluster I were found to have the highest biological activities, in particular the antioxidant properties, anti-tyrosinase, anti-amylase and anti-BChE activities. Well over the maximum amount of TPC and TFvl were achieved by the members of this clusters. Interestingly, this clusters were composed exclusively of the stem bark extracts of both species the majority of which were obtained using the methanol as solvent. This finding revealed that the stem bark of both species is more active than the leaves. It also suggested that the stem bark is richer in polar polyphenols. Sinan et al. [61] reported variation of antioxidant and enzyme inhibitory activities between the leaves and stem bark of *U. togoensis* and *C. procera.* Many authors argued that the concentration of molecules in plants tissues and/or parts vary considerably in terms of its ontogenetic development, the activity of some enzymes and under the impact of soil nutrient and environmental conditions [61,62]. Thereby, the biological activity of the extract of any plant being tightly bound to quantity of quality of molecules it contains, the ideal part should be chosen to derive the full potential of said plant. Furthermore, as evidenced that the stem bark of both species contained highest level of phenolic compounds and since the solubility of phenolic compounds depend on the present and position of –OH groups and the molecular size and the length of constituent hydrocarbon chains [63], it would be reasonable to use an intermediate polar solvent for the future investigations on these two species. The effectiveness of polar solvent like methanol is due to its intermediate polarity that allows it to easily dissolve low molecular weight having protonatable functional groups, i.e., OH [64]. However, considering the toxicity of methanol solvent for human, it would be preferred to evaluate other non-toxic solvent, i.e., ethanol, which possess polarity close to that of methanol.

**Figure 7:**
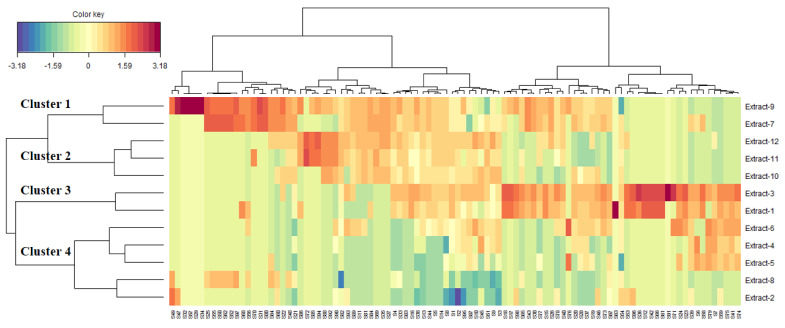
Cluster image map (CIM) analysis of the chemical composition data of *Spondias* species (Red color: high concentration, Blue color: low concentration). Extract 1-*Spondias dulcis*-leaves-Infusion, Extract 2-*S. dulcis*-leaves-MAC (no stir)-EA; Extract 3-*S. dulcis*-leaves-MAC (no stir)-MEOH; Extract 4-*S. dulcis*-stem bark-infusion; Extract 5-*S. dulcis*-stem bark-MAC (no stir)-EA; Extract 6-*S. dulcis*-stem bark-MAC (no stir)-MEOH; Extract 7-*Spondias mombin* leaves-infusion; Extract 8- *S. mombin* leaves-MAC (no stir) EA; Extract 9-*S. mombin*-leaves-MAC (no stir)- MEOH; Extract 10-*S. mombin*-stem bark-Infusion; Extract 11-*S. mombin*-stem bark-MAC (no stir)-EA; Extract 12-*S. mombin*-stem bark-MAC (no stir)-MEOH.). For compounds numbers refer to Table 1.

The consolidation of the 12 samples according to the amount of identified secondary metabolites was shown in Figure 7. As can be seen, the samples can be separated in four main cluster. Samples included in the cluster I (*Spondias mombin* leaves-infusion and *S. mombin*-leaves-MAC (no stir)) were richer in many compounds following by that of the cluster 3 (1-*Spondias dulcis*-leaves-Infusion and 3-*S. dulcis*-leaves-MAC (no stir)-MEOH) and cluster 2 (*S. mombin*-stem bark-Infusion; *S. mombin*-stem bark-MAC (no stir)-EA, and *S. mombin*-stem bark-MAC (no stir)-MEOH). The samples constituting the cluster 4 were lower in the majority of identified compounds. Moreover, some extracts were, individually, remarkably rich in many compounds. Illustratively, *Spondias mombin* leaves-infusion was rich in galloyl-geraniin (S29), ellagic acid- pentoside isomer (S32), ellagic acid-pentoside (S34), caffeic acid *O*-hexoside (S47), and kaempferol-3-*O*-dihexoside (S57). Similarly, ellagic acid deoxyhexoside (S33) was abundant in *Spondias dulcis*-leaves-Infusion while 3-*S. cythera*-leaves-MAC (no stir)-MEOH was rich in di HHDP-hexoside (S36), myricetin 7-*O*-deoxyhexoside (S71), and naringenin 8-*C*-hexoside (S91).

## 4. Conclusions

Data presented in this study highlight the antioxidant and enzyme inhibitory potential of *S. mombin* and *S. dulcis*. It was also observed that the type of extraction solvent affects the extraction of bioactive secondary metabolites which subsequently determine the biological activity of the herbal extract. Extracts of *S. mombin* and *S. dulcis* leaves and stem bark obtained by organic solvent showed potent tyrosinase, acetylcholinesterase, and α-glucosidase inhibition while relatively low inhibition was observed against butyrylcholinesterase and α-amylase. It was also noted that methanol was a good extracting solvent of phenolics, which was in turn associated with high radical scavenging and reducing properties. Ellagic and gallic acid derivatives, and ellagitannins together with flavonols and flavanones could be associated with the prospective biological activity of stem bark extracts. The selected *Spondias* species could be considered as valuable sources of bioactive compounds in the pharmaceutical, cosmeceutical, and nutraceutical applications.

## Figures and Tables

**Table 1:** Specialized metabolites in *Spondias* extracts assayed by UHPLC-ESI-MS/MS.

No	Identified/Tentatively Annotated Compound	Molecular Formula	Exact Mass [M-H]^−^	Distribution
Gallic acid and galloyl derivatives				
1.	galloyl hexose	C_13_H_16_O_10_	331.0671	1,2,3,4,5,6,7,8,9,10,11,12
2.	galloyl hexose isomer	C_13_H_16_O_10_	331.0671	1,3,4,5,6,7,8,9,10,11,12
3.	gallic acid *	C_7_H_6_O_5_	169.0142	1,2,3,4,5,6,7,8,9,10,11,12
4.	gallic acid hexoside	C_13_H_16_O_10_	331.0671	1,3,7,8,9,10,11,12
5.	digalloyl hexoside	C_20_H_20_O_14_	483.0780	1,3,6,7,9,10,11,12
6.	digalloylquinic acid	C_21_H_20_O_14_	495.0776	1,3,4,5,6,7
7.	digalloylquinic acid isomer	C_21_H_20_O_14_	495.0790	1,3,4,5,6
8.	digalloyl hexose	C_20_H_20_O_14_	483.0780	1,3,5,6,7,8,9,10,11,12
9.	methylgallate	C_8_H_8_O_5_	183.0289	1,2,3,4,5,6,7,8,9,10,11,12
10.	epigallocatechin-*O*-gallate	C_22_H_18_O_11_	457.0776	1,2,3,4,5,6,7,8,9,10,11,12
11.	trigalloyl hexoside	C_27_H_24_O_18_	635.0890	6,7,8,9,10,11,12
12.	dimethylgallate	C_9_H_10_O_5_	197.0455	1,2,3
13.	trigalloyl hexoside isomer	C_27_H_24_O_18_	635.0890	1,3,4,6,7,9,10,11,12
14.	tetragalloyl hexoside	C_34_H_28_O_22_	787.0999	1,3,4,6,7,9,10,11,12
Ellagic acid derivatives and ellagitannins				
15.	HHDP-hexoside	C_20_H_18_O_14_	481.0624	1,3,7,9,12
16.	HHDP-hexoside isomer	C_20_H_18_O_14_ isomer	481.0624	1,3,7,9,10,12
17.	galloyl-HHDP-hexoside	C_27_H_22_O_18_	633.0733	1,3,8,9
18.	galloyl-HHDP-hexoside isomer	C_27_H_22_O_18_	633.0733	1,3,7,9
19.	galloyl-HHDP-hexoside isomer	C_27_H_22_O_18_	633.0733	1,3,5,6,7,9,10,12
20.	brevifolin carboxylic acid	C_13_H_8_O_8_	291.0149	1,3,4,6,7,9,10
21.	galloyl-HHDP-hexoside isomer	C_27_H_22_O_18_	633.0733	1,3,4,6,7,8,9,10,11,12
22.	geraniin	C_41_H_28_O_27_	951.0745	3,7,10,12
23.	valoneic acid-dilactone	C_21_H_10_O_13_	469.0049	1,3,6
24.	digalloyl-HHDP-hexoside	C_34_H_26_O_22_	785.0843	1,3,5,6
25.	ellagic acid-hexoside	C_20_H_16_O_13_	463.0518	7,8,9
26.	brevifolin	C_12_H_8_O_6_	247.0245	1,3,7,9,12
27.	digalloyl-HHDP-hexoside isomer	C_34_H_26_O_22_	785.0843	7,9,10,11,12
28.	methylellagic acid-*O*-hexoside	C_21_H_18_O_13_	477.0675	1,3,4,5,6,7,9,10
29.	galloyl-geraniin	C_48_H_32_O_31_	1103.0855	9
30.	methyl brevifolin carboxylate	C_14_H_10_O_8_	305.0304	1,3,7,9
31.	galloyl-bisHHDP-hexoside	C_41_H_30_O_27_	953.0902	7,9
32.	ellagic acid- pentoside isomer	C_19_H_14_O_12_	433.0412	9
33.	ellagic acid deoxyhexoside	C_20_H_16_O_12_	447.0571	1,3,7,8,9,10,11,12
34.	ellagic acid- pentoside	C_19_H_14_O_12_	433.0412	9
35.	digalloyl-HHDP-hexoside isomer	C_34_H_26_O_22_	785.0843	7,9,10,11,12
36.	di HHDP-hexoside	C_34_H_24_O_22_	783.0686	1,3
37.	methylellagic acid *O*-hexoside	C_21_H_18_O_13_	477.0675	1,3,4,5,6,7,9,10
38.	ellagic acid *	C_14_H_6_O_8_	300.9991	1,2,3,4,5,6,7,8,9,10,11,12
39.	dimethylellagic acid *O*-hexoside	C_22_H_20_O_13_	491.0831	1,2,3,4,5,6,7
40.	methylellagic acid	C_15_H_8_O_8_	315.0146	1,3,6,7,9,12
41.	dimethylellagic acid *O*-hexoside	C_22_H_20_O_13_	491.0831	1,3,4,5,6
42.	trimethylellagic acid	C_17_H_12_O_8_	343.0459	1,3
43.	dimethylellagic acid	C_16_H_10_O_8_	329.0303	1,3,7,9
Hydroxybenzoic, hydroxycinnamic, acylquinic acids and derivatives				
44.	protocatechuic acid *O*-hexoside	C_13_H_16_O_9_	315.0723	1,3,6,7,9,10,11,12
45.	protocatechuic acid *	C_7_H_6_O_4_	153.0181	1,2,3,4,5,6,7,8,9,10,11,12
46.	syringic acid *O*-hexoside	C_15_H_20_O_10_	359.0956	1,2,3,6,7,9,10
47.	caffeic acid *O*-hexoside	C_15_H_18_O_9_	341.0883	9
48.	neochlorogenic acid *	C_16_H_18_O_9_	353.0878	7,8,9,10,11,12
49.	caffeic acid *	C_9_H_8_O_4_	179.0342	8,9
50.	chlorogenic acid *	C_16_H_18_O_9_	353.0877	7,8,9
51.	vanillic acid *O*-hexoside	C_14_H_18_O_9_	329.0888	1,3,4,5,6,7,10,11,12
52.	4-caffeoylquinic aid	C_16_H_18_O_9_	353.0880	7,8
53.	syringic acid *	C_9_H_10_O_5_	197.0447	1,3,12
54.	salicylic acid	C_7_H_6_O_3_	137.0231	1,2,3,4,5,6,7,8,9,10,11,12
Flavonols				
55.	quercetin 3-*O*-dihexoside	C_27_H_30_O_17_	625.1415	7,8,9
56.	quercetin 3-*O*-hexoside-*O*-pentoside	C_26_H_28_O_16_	595.1307	1,7,8,9
57.	kaempferol 3-*O*-dihexoside	C_27_H_30_O_16_	609.1436	9
58.	myricetin 3-*O*-pentoside	C_20_H_18_O_12_	449.0727	1,3,5,7
59.	myricitrin (myricetin 3-*O*-rhamnoside) *	C_21_H_20_O_12_	463.0882	1,3,4,5,6
60.	isoquercitrin (quercetin 3-*O*-glucoside) *	C_21_H_20_O_12_	463.0883	7,8,9,10,12
61.	myricetin 3-*O*-hexoside-7-*O*-deoxyhexoside	C_27_H_30_O_17_	625.1418	1,3
62.	kaempferol 3-*O*-pentosyl-hexoside	C_26_H_28_O_15_	579.1359	7,8,9
63.	quercetin 4′-*O*-hexuronide	C_21_H_18_O_13_	477.0680	1
64.	hyperoside (quercetin 3-*O*-galactoside) *	C_21_H_20_O_12_	463.0885	5,7,8,9,12
65.	quercetin-*O*-pentoside	C_20_H_18_O_11_	433.0776	1,3,7,8,9,10,11
66.	methylmyricetin-*O*-deoxyhexoside	C_22_H_22_O_12_	477.1038	1,3
67.	kaempferol 3-*O*-glucoside *	C_21_H_20_O_11_	447.0933	1,3,5,6,7,8,9
68.	quercitrin (quercetin 3-*O*-rhamnoside) *	C_21_H_20_O_11_	447.0933	1,6,7,9
69.	kaempferol 7-*O*-glucoside	C_21_H_20_O_11_	447.0941	7,9,10,11,12
70.	quercetin 4′-*O*-hexoside	C_21_H_20_O_12_	463.0892	7,9,11
71.	myricetin 7-*O*-deoxyhexoside	C_21_H_20_O_12_	463.0885	3,5,6
72.	isorhamnetin 3-O-glucoside *	C_22_H_22_O_12_	477.1042	11,12
73.	kaempferol-*O*-pentoside	C_20_H_18_O_10_	417.0828	1,3,6,7,9
74.	myricitrin-*O*-gallate	C_28_H_24_O_16_	615.0999	1,3,5,6
75.	kaempferol 3-O-deoxyhexoside	C_21_H_20_O_10_	431.0985	1,3,4,5,6
76.	rhamnetin 3-*O*-hexoside	C_22_H_22_O_12_	477.1035	5,6,9
77.	Quercetin *	C_15_H_10_O_7_	301.0347	1,3,5,7,9,11,12
78.	Myricetin *	C_15_H_10_O_8_	317.0301	1,3,6,7,9,11,12
79.	rhamnetin 3-*O*-deoxyhexoside	C_22_H_22_O_11_	461.1090	3,4,5,6
80.	Kaempferol *	C_15_H_10_O_6_	285.0407	7,9
Flavanones, flavanonols and flavan-3-ols				
81.	(epi)catechin *O*-hexoside	C_21_H_24_O_11_	451.1241	6,7,9,10,11,12
82.	(+)-catechin *	C_15_H_14_O_6_	289.0717	1,3,4,5,6,7,9,10,11,12
83.	taxifolin 3-*O*-hexoside	C_21_H_22_O_12_	465.1039	1,3,7,9,10,11,12
84.	taxifolin 7-*O*-hexoside	C_21_H_22_O_12_	465.1037	1,7,9,10,11,12
85.	(epi)catechin-gallate	C_22_H_18_O_10_	441.0828	9,11,12
86.	taxifolin 4′-*O*-hexoside	C_21_H_22_O_12_	465.1037	1,7,9,11,12
87.	naringenin 7-*O*-glucoside (prunin) *	C_21_H_22_O_10_	433.1140	1,3,4,5,6,9,10,11,12
88.	(epi)catechin-gallate isomer I	C_22_H_18_O_10_	441.0826	9,10,11,12
89.	eriodictiol 7-*O*-hexoside	C_21_H_22_O_11_	449.1090	3,6,7,9,10,11,12
90.	(epi)catechin-gallate isomer II	C_22_H_18_O_10_	441.0826	1,4,6,9,10,11,12
91.	naringenin 8-*C*-hexoside	C_21_H_22_O_10_	433.1144	3
92.	pinocembrin-O-hexoside	C_21_H_22_O_9_	417.1195	9,10,11,12
93.	naringenin	C_15_H_12_O_5_	271.0614	3,5,6,7,9,11,12
94.	pinocembrin	C_15_H_12_O_4_	255.0664	9,11,12
Others				
95.	sucrose	C_12_H_22_O_11_	341.1088	1,2,3,4,5,6,7,8,9,10,11,12
96.	tuberonic acid-hexoside	C_18_H_28_O_9_	387.1657	1,3
97.	cinchonain I	C_24_H_20_O_9_	451.1037	7,8,9
98.	pinoresinol (Eklund et al., 2008)	C_20_H_22_O_6_	357.1345	1,3,9

*-compare to reference standard; 1-*Spondias dulcis*-leaves-Infusion, 2-*S. dulcis*-leaves-MAC (no stir)-EA; 3-*S. dulcis*-leaves-MAC (no stir)-MEOH; 4-*S. dulcis*-stem bark-infusion; 5-*S. dulcis*-stem bark-MAC (no stir)-EA; 6-*S. dulcis*-stem bark-MAC (no stir)-MEOH; 7-*Spondias mombin* leaves-infusion; 8-*S. mombin* leaves-MAC (no stir) EA; 9-*S. mombin*-leaves-MAC (no stir)-MEOH; 10-*S. mombin*-stem bark-Infusion; 11-*S. mombin*-stem bark-MAC (no stir)-EA; 12-*S. mombin*-stem bark-MAC (no stir)-MEOH.

**Table 2:** Enzyme inhibitory effects of the tested extracts.

Species	Parts	Methods-Solvents	AChE Inhibition (mg GALAE/g)	BChE Inhibition (mg GALAE/g)	Tyrosinase Inhibition (mg KAE/g)	Amylase Inhibition (mmol ACAE/g)	Glucosidase Inhibition (mmol ACAE/g)
*Spondias dulcis*	Leaves	Infusion	10.10 ± 0.11 ^ab^	3.83 ± 0.48 ^cdefg^	na	0.25 ± 0.04 ^n^	na
		Maceration-EA	8.60 ± 0.23 ^bcdef^	na	82.27 ± 8.84 ^n^	0.71 ± 0.01 ^efghi^	11.07 ± 0.21 ^d^
		Maceration-MeOH	9.79 ± 0.79 ^abcd^	3.44 ± 0.25 ^cdefg^	165.44 ± 2.39 ^d^	0.88 ± 0.03 ^c^	17.37 ± 0.11 ^a^
		Maceration-EA (not stir)	5.77 ± 0.66 ^h^	6.21 ± 0.18 ^ab^	94.17 ± 5.97 ^m^	0.76 ± 0.01 ^de^	14.73 ± 0.74 ^bc^
		Maceration-MeOH (not stir)	6.89 ± 0.21 ^gh^	1.98 ± 0.44 ^ghi^	184.49 ± 2.51 ^c^	0.97 ± 0.03 ^a^	17.70 ± 0.04 ^a^
		Soxhlet-EA	9.51 ± 0.99 ^abcd^	1.21 ± 0.44 ^hi^	79.88 ± 2.02 ^n^	0.54 ± 0.01 ^l^	14.27 ± 2.96 ^c^
		Soxhlet-MeOH	8.82 ± 0.25 ^abcde^	2.55 ± 0.72 ^efgh^	178.97 ± 1.28 ^c^	0.80 ± 0.01 ^d^	17.61 ± 0.03 ^a^
	Stem barks	Infusion	9.95 ± 0.53 ^abcd^	3.36 ± 0.68 ^cdefgh^	15.72 ± 1.90 ^o^	0.32 ± 0.01 ^m^	na
		Maceration-EA	10.33 ± 1.09 ^a^	3.62 ± 0.39 ^cdefg^	119.85 ± 1.47 ^k^	0.70 ± 0.01 ^efghi^	16.88 ± 0.02 ^a^
		Maceration-MeOH	7.81 ± 0.62 ^efg^	4.83 ± 0.14 ^abcd^	197.72 ± 1.43 ^b^	0.99 ± 0.01 ^a^	na
		Maceration-EA (not stir)	9.58 ± 0.77 ^abcd^	3.68 ± 1.49 ^cdefg^	116.15 ± 3.31 ^k^	0.67 ± 0.01 ^hij^	16.94 ± 0.11 ^a^
		Maceration-MeOH (not stir)	8.39 ± 0.19 ^cdefg^	5.09 ± 0.08 ^abc^	201.48 ± 1.17 ^ab^	0.94 ± 0.01 ^abc^	na
		Soxhlet-EA	9.98 ± 0.23 ^abc^	3.55 ± 0.37 ^cdefg^	155.95 ± 3.27 ^efg^	0.71 ± 0.04 ^efgh^	16.91 ± 0.03 ^a^
		Soxhlet-MeOH	7.00 ± 0.14 ^fgh^	5.39 ± 0.72 ^abc^	198.93 ± 0.85 ^ab^	0.90 ± 0.01 ^bc^	na
*Spondias mombin*	Leaves	Infusion	10.45 ± 0.16 ^a^	2.54 ± 0.56 ^efgh^	na	0.13 ± 0.01 ^o^	17.12 ± 0.13 ^a^
		Maceration-EA	8.66 ± 0.77 ^bcde^	2.88 ± 0.52 ^defgh^	140.14 ± 2.50 ^i^	0.69 ± 0.04 ^fghi^	16.20 ± 0.13 ^ab^
		Maceration-MeOH	10.01 ± 0.13 ^abc^	4.48 ± 0.21 ^bcde^	159.58 ± 1.37 ^def^	0.55 ± 0.01 ^l^	na
		Maceration-EA (not stir)	7.77 ± 0.97 ^efg^	2.17 ± 0.87 ^fgh^	129.52 ± 1.19 ^j^	0.73 ± 0.02 ^efg^	16.23 ± 0.50 ^ab^
		Maceration-MeOH (not stir)	8.59 ± 0.45 ^bcdef^	6.89 ± 1.95 ^a^	153.70 ± 0.62 ^fgh^	0.62 ± 0.01 ^jk^	17.41 ± 0.19 ^a^
		Soxhlet-EA	8.33 ± 0.65 ^defg^	4.15 ± 0.44 ^bcdef^	147.24 ± 1.93 ^hi^	0.68 ± 0.02 ^fghij^	16.79 ± 0.40 ^a^
		Soxhlet-MeOH	10.37 ± 0.07 ^a^	4.32 ± 0.94 ^bcde^	156.41 ± 1.05 ^efg^	0.57 ± 0.01 ^kl^	na
	Stem barks	Infusion	9.93 ± 0.04 ^abcd^	3.71 ± 0.34 ^cdefg^	104.08 ± 1.11 ^l^	0.65 ± 0.04 ^ij^	na
		Maceration-EA	9.27 ± 0.03 ^abcde^	2.89 ± 1.03 ^defgh^	150.72 ± 1.82 ^gh^	0.68 ± 0.03 ^ghij^	16.96 ± 0.04 ^a^
		Maceration-MeOH	9.34 ± 0.21 ^abcde^	5.26 ± 0.57 ^abc^	203.72 ± 1.02 ^ab^	0.94 ± 0.03 ^abc^	na
		Maceration-EA (not stir)	10.31 ± 0.47 ^a^	1.97 ± 0.19 ^ghi^	146.82 ± 0.19 ^hi^	0.74 ± 0.01 ^def^	16.82 ± 0.03 ^a^
		Maceration-MeOH (not stir)	8.59 ± 0.12 ^bcdef^	4.51 ± 0.30 ^bcde^	207.00 ± 0.73 ^a^	0.95 ± 0.01 ^ab^	na
		Soxhlet-EA	9.39 ± 0.39 ^abcde^	6.28 ± 0.33 ^ab^	163.23 ± 2.54 ^de^	0.72 ± 0.01 ^efgh^	17.16 ± 0.03 ^a^
		Soxhlet-MeOH	9.02 ± 0.14 ^abcde^	4.36 ± 0.09 ^bcde^	na	0.96 ± 0.01 ^ab^	na

Values are reported as mean ± S.D. GALAE: Galantamine equivalent; KAE: Kojic acid equivalent; ACAE: Acarbose equivalent; nd: not detected. Different letters (ao) indicate significant differences in the tested extracts (*p* < 0.05).

**Table 3:** Total bioactive compounds (phenolic (TPC), flavonoid (TFC), phenolic acid (TPAC), and flavonol (TFlv) and total antioxidant capacity (by phosphomolybdenum assay) of the tested extracts.

Species	Parts	Methods-Solvents	TPC (mg GAE/g)	TFC (mg RE/g)	TPAC (mg CAE/g)	TFlv (mg CE/g)	Phosphomolybdenum (mmol TE/g)
*Spondias dulcis*	Leaves	Infusion	179.89 ± 0.14 ^ef^	25.30 ± 0.59 ^d^	6.91 ± 0.74 ^h^	0.46 ± 0.01 ^k^	4.89 ± 0.14 ^efg^
		Maceration-EA	29.27 ± 0.91 ^qr^	13.80 ± 0.49 ^g^	nd	1.82 ± 0.06 ^jk^	3.33 ± 0.14 ^k^
		Maceration-MeOH	134.39 ± 1.96 ^j^	43.41 ± 1.08 ^a^	nd	7.35 ± 0.04 ^gh^	3.94 ± 0.12 ^hijk^
		Maceration-EA (not stir)	32.60 ± 0.32 ^pq^	13.21 ± 0.59 ^g^	2.63 ± 0.22 ^ij^	2.33 ± 0.02 ^jk^	3.49 ± 0.14 ^ijk^
		Maceration-MeOH (not stir)	182.53 ± 1.15 ^e^	43.11 ± 3.28 ^a^	nd	4.85 ± 0.05 ^hij^	4.98 ± 0.02 ^ef^
		Soxhlet-EA	25.85 ± 0.97 ^r^	21.26 ± 0.26 ^e^	nd	1.22 ± 0.01 ^jk^	1.75 ± 0.12 ^l^
		Soxhlet-MeOH	136.96 ± 1.86 ^ij^	40.71 ± 3.63 ^a^	nd	9.48 ± 0.01 ^fg^	3.87 ± 0.25 ^hijk^
	Stem barks	Infusion	143.60 ± 0.90 ^h^	8.53 ± 0.43 ^h^	4.28 ± 0.52 ^i^	1.32 ± 0.01 ^jk^	4.27 ± 0.09 ^fgh^
		Maceration-EA	47.28 ± 1.02 ^o^	4.42 ± 0.22 ^ijk^	nd	7.48 ± 0.05 ^gh^	2.16 ± 0.08 ^l^
		Maceration-MeOH	230.08 ± 3.14 ^b^	2.30 ± 0.20 ^kl^	nd	12.52 ± 0.17 ^def^	6.82 ± 0.28 ^cd^
		Maceration-EA (not stir)	44.19 ± 0.93 ^o^	5.85 ± 0.08 ^hi^	3.77 ± 0.81 ^ij^	6.24 ± 0.13 ^ghi^	2.31 ± 0.11 ^l^
		Maceration-MeOH (not stir)	240.24 ± 1.08 ^a^	2.40 ± 0.09 ^kl^	nd	14.27 ± 0.07 ^cde^	7.77 ± 0.18 ^b^
		Soxhlet-EA	92.69 ± 0.99 ^m^	6.47 ± 0.23 ^hi^	nd	18.95 ± 0.47 ^b^	3.43 ± 0.27 ^jk^
		Soxhlet-MeOH	216.16 ± 1.00 ^c^	2.47 ± 0.07 ^jkl^	nd	11.66 ± 0.02 ^ef^	6.24 ± 0.70 ^d^
*Spondias mombin*	Leaves	Infusion	143.12 ± 1.00 ^hi^	12.14 ± 0 11 ^g^	27.16 ± 0.64 ^a^	0.60 ± 0.01 ^k^	3.65 ± 0.14 ^hijk^
		Maceration-EA	46.37 ± 0.41 ^o^	17.06 ± 0.33 ^f^	nd	2.81 ± 0.02 ^ijk^	3.52 ± 0.29 ^hijk^
		Maceration-MeOH	174.93 ± 1.00 ^f^	29.02 ± 0.69 ^c^	16.87 ± 0.51 ^d^	2.09 ± 0.02 ^jk^	4.18 ± 0.08 ^ghij^
		Maceration-EA (not stir)	36.97 ± 0.98 ^p^	13.51 ± 0.34 ^g^	nd	2.23 ± 0.03 ^jk^	3.85 ± 0.18 ^hijk^
		Maceration-MeOH (not stir)	112.14 ± 0.24 ^l^	27.51 ± 0.12 ^cd^	9.64 ± 0.56 ^g^	8.78 ± 0.08 ^fg^	4.26 ± 0.21 ^fghi^
		Soxhlet-EA	59.54 ± 0.24 ^n^	5.58 ± 0.37 ^hij^	2.43 ± 0.77 ^j^	3.04 ± 0.01 ^ijk^	3.76 ± 0.18 ^hijk^
		Soxhlet-MeOH	151.20 ± 1.55 ^g^	33.16 ± 0.15 ^b^	14.76 ± 0.07 ^ef^	3.31 ± 0.03 ^ijk^	4.29 ± 0.13 ^fgh^
	Stem barks	Infusion	189.85 ± 1.25 ^d^	3.39 ± 0.09 ^ijkl^	23.25 ± 1.18 ^b^	0.85 ± 0.02 ^k^	5.34 ± 0.09 ^e^
		Maceration-EA	107.43 ± 1.35 ^l^	1.21 ± 0.14 ^l^	14.50 ± 0.91 ^f^	25.83 ± 6.22 ^a^	3.71 ± 0.07 ^hijk^
		Maceration-MeOH	245.50 ± 3.20 ^a^	1.41 ± 0.11 ^kl^	15.22 ± 0.23 ^def^	15.48 ± 0.29 ^bcd^	7.20 ± 0.30 ^bc^
		Maceration-EA (not stir)	119.57 ± 4.83 ^k^	1.26 ± 0.04 ^kl^	19.32 ± 1.40 ^c^	18.50 ± 0.39 ^b^	4.05 ± 0.22 ^hijk^
		Maceration-MeOH (not stir)	244.28 ± 5.42 ^a^	1.77 ± 0.21 ^kl^	27.54 ± 0.73 ^a^	18.13 ± 0.43 ^b^	8.76 ± 0.49 ^a^
		Soxhlet-EA	156.46 ± 1.60 ^g^	1.94 ± 0.04 ^kl^	20.12 ± 0.93 ^c^	18.03 ± 0.41 ^bc^	4.89 ± 0.18 ^efg^
		Soxhlet-MeOH	228.18 ± 2.31 ^b^	1.55 ± 0.07 ^kl^	16.44 ± 0.29 ^de^	17.68 ± 0.05 ^bc^	6.50 ± 0.34 ^cd^

Values are reported as mean ± S.D. GAE: Gallic acid equivalent; RE: Rutin equivalent; CAE: Caffeic acid equivalent; CE: Catechin equivalent: TE: Trolox equivalent; nd: not detected. Different letters (aq) indicate significant differences in the tested extracts (*p* < 0.05).

**Table 4:** Antioxidant properties of the tested extracts.

Species	Parts	Methods-Solvents	DPPH (mg TE/g)	ABTS (mg TE/g)	CUPRAC (mg TE/g)	FRAP (mg TE/g)	Metal Chelating (mg EDTAE/g)
*Spondias dulcis*	Leaves	Infusion	619.35 ± 1.10 ^b^	1115.63 ± 3.70 ^f^	1109.52 ± 7.96 ^f^	687.23 ± 5.19 ^f^	30.97 ± 2.15 ^def^
		Maceration-EA	17.82 ± 2.41 ^jk^	29.11 ± 6.59 ^p^	104.60 ± 1.36 ^q^	37.64 ± 0.64 ^o^	34.78 ± 1.64 ^cd^
		Maceration-MeOH	259.85 ± 0.14 ^e^	658.05 ± 0.53 ^j^	733.72 ± 22.82 ^j^	405.48 ± 4.73 ^j^	19.78 ± 0.31 ^lm^
		Maceration-EA (not stir)	16.23 ± 1.92 ^k^	21.21 ± 0.17 ^p^	122.46 ± 2.02 ^q^	45.69 ± 1.21 ^no^	25.67 ± 0.67 ^ghi^
		Maceration-MeOH (not stir)	609.71 ± 1.58 ^b^	1076.92 ± 6.96 ^g^	1107.96 ± 16.31 ^f^	632.18 ± 5.99 ^g^	17.58 ± 0.97 ^mn^
		Soxhlet-EA	28.51 ± 2.58 ^j^	15.98 ± 4.21 ^p^	93.71 ± 3.86 ^q^	47.77 ± 1.24 ^no^	10.02 ± 0.37 ^pq^
		Soxhlet-MeOH	261.69 ± 0.25 ^e^	662.37 ± 0.81 ^j^	884.09 ± 12.38 ^h^	502.83 ± 5.24 ^i^	14.64 ± 1.08 ^no^
	Stem barks	Infusion	252.51 ± 0.37 ^e^	660.88 ± 0.86 ^j^	810.96 ± 7.10 ^i^	494.47 ± 7.75 ^i^	24.68 ± 0.83 ^hijk^
		Maceration-EA	117.97 ± 1.11 ^gh^	180.04 ± 1.64 ^m^	204.32 ± 4.55 ^o^	97.41 ± 3.58 ^mn^	5.77 ± 0.56 ^r^
		Maceration-MeOH	657.31 ± 0.45 ^a^	1601.11 ± 10.38 ^b^	1567.07 ± 27.85 ^d^	954.81 ± 28.93 ^c^	21.45 ± 2.88 ^jklm^
		Maceration-EA (not stir)	107.54 ± 0.81 ^h^	156.72 ± 2.25 ^mn^	186.75 ± 2.23 ^o^	96.84 ± 1.94 ^mn^	na
		Maceration-MeOH (not stir)	656.25 ± 0.54 ^a^	1657.40 ± 1.72 ^a^	1787.56 ± 13.10 ^b^	1065.65 ± 23.89 ^b^	20.80 ± 0.67 ^klm^
		Soxhlet-EA	131.03 ± 0.13 ^f^	330.61 ± 0.12 ^l^	409.34 ± 13.29 ^m^	218.78 ± 13.82 ^l^	11.89 ± 0.56 ^opq^
		Soxhlet-MeOH	657.27 ± 0.59 ^a^	1479.83 ± 7.59 ^d^	1309.52 ± 12.49 ^e^	814.43 ± 27.99 ^d^	20.41 ± 1.40 ^lm^
*Spondias mombin*	Leaves	Infusion	254.63 ± 2.05 ^e^	628.26 ± 4.35 ^k^	664.65 ± 4.24 ^k^	490.70 ± 2.10 ^i^	21.86 ± 0.63 ^ijkl^
		Maceration-EA	59.66 ± 2.18 ^i^	89.25 ± 4.67 ^o^	178.94 ± 3.24 ^op^	71.95 ± 2.74 ^mno^	31.71 ± 0.94 ^de^
		Maceration-MeOH	570.90 ± 14.85 ^c^	944.48 ± 12.25 ^i^	955.54 ± 29.11 ^g^	613.45 ± 24.53 ^gh^	27.27 ± 0.40 ^fgh^
		Maceration-EA (not stir)	24.61 ± 1.25 ^jk^	21.50 ± 2.48 ^p^	137.66 ± 6.38 ^pq^	52.94 ± 0.56 ^no^	45.19 ± 0.93 ^a^
		Maceration-MeOH (not stir)	125.91 ± 0.06 ^fg^	329.50 ± 0.28 ^l^	443.61 ± 33.81 ^m^	225.48 ± 29.90 ^l^	40.40 ± 1.59 ^b^
		Soxhlet-EA	65.04 ± 1.99 ^i^	133.21 ± 7.15 ^n^	261.95 ± 3.73 ^n^	112.77 ± 1.64 ^m^	25.09 ± 2.85 ^hij^
		Soxhlet-MeOH	258.87 ± 0.20 ^e^	658.11 ± 0.21 ^j^	776.86 ± 12.30 ^ij^	466.31 ± 4.46 ^i^	36.54 ± 1.01 ^bc^
	Stem barks	Infusion	528.64 ± 2.15 ^d^	1009.25 ± 9.40 ^h^	1118.43 ± 6.39 ^f^	749.16 ± 3.86 ^e^	29.51 ± 1.14 ^efg^
		Maceration-EA	130.13 ± 0.22 ^f^	331.05 ± 0.27 ^l^	527.95 ± 3.27 ^l^	299.55 ± 2.14 ^k^	8.46 ± 1.68 ^qr^
		Maceration-MeOH	657.82 ± 0.32 ^a^	1584.71 ± 24.46 ^bc^	1672.05 ± 20.01 ^c^	1030.53 ± 22.80 ^b^	18.49 ± 1.56 ^lmn^
		Maceration-EA (not stir)	131.46 ± 0.40 ^f^	331.55 ± 0.14 ^l^	627.77 ± 23.13 ^k^	375.28 ± 10.25 ^j^	na
		Maceration-MeOH (not stir)	660.19 ± 1.18 ^a^	1659.38 ± 1.31 ^a^	2123.67 ± 28.84 ^a^	1379.24 ± 35.00 ^a^	12.80 ± 1.14 ^op^
		Soxhlet-EA	560.05 ± 10.56 ^c^	1219.18 ± 6.47 ^e^	180.16 ± 5.12 ^op^	572.17 ± 37.58 ^h^	9.26 ± 0.61 ^pqr^
		Soxhlet-MeOH	656.01 ± 0.63 ^a^	1566.78 ± 22.64 ^c^	285.80 ± 4.55 ^n^	845.72 ± 16.88 ^d^	13.05 ± 1.04 ^op^

Values are reported as mean ± S.D. GAE: TE: Trolox equivalent; EDTAE: EDTA equivalent. Different letters (aq) indicate significant differences in the tested extracts (*p* < 0.05).

## Data Availability

Data is contained within the article and Supplementary Materials.

## Supplementary Materials

The following are available online at https://www.mdpi.com/article/10.3390/antiox10111771/s1, Table S1. Eigen values and percentage of explained variance of each principal component (PC).
