# Supplementary material for: Exploring the Chemical Profiles and Biological Values of Two Spondias Species (S. dulcis and S. mombin): Valuable Sources of Bioactive Natural Products
Source: Antioxidants (Basel). 2021 Nov 5;10(11):1771. doi: 10.3390/antiox10111771 (PMC8614698; doi:10.3390/antiox10111771)
Supplement: Supplementary file 1 [file antioxidants-10-01771-s001.zip › antioxidants-1439531-supplementary.pdf]

Article

Exploring the Chemical Profiles and Biological Values of Two *Spondias* Species (*S. dulcis* and *S. mombin*): Valuable Sources of Bioactive Natural Products

Kouadio Ibrahime Sinan [1], Gokhan Zengin [1,*], Dimitrina Zheleva-Dimitrova [2], Reneta Gevrenova [2], Marie Carene Nancy Picot-Allain [3], Stefano Dall'Acqua [4,*], Tapan Behl [5], Bey Hing Goh [6,7], Patrick Tang Siah Ying [8] and Mohamad Fawzi Mahomoodally [3,*]

1 Biochemistry and Physiology Research Laboratory, Department of Biology, Science Faculty, Selcuk University, Konya 42130, Turkey; sinankouadio@gmail.com

2 Department of Pharmacognosy, Faculty of Pharmacy, Medical University—Sofia, 2 Dunav Str., 1000 Sofia, Bulgaria; dzheleva@pharmfac.mu-sofia.bg (D.Z.-D.); rgevrenova@pharmfac.mu-sofia.bg (R.G.)

3 Department of Health Sciences, Faculty of Medicine and Health Sciences, University of Mauritius, Réduit 80837, Mauritius; picotcarene@yahoo.com

4 Department of Pharmaceutical and Pharmacological Sciences, University of Padova, Via Marzolo 5, 35131 Padova, Italy

5 Chitkara College of Pharmacy, Chitkara University, Punjab 140401, India; tapanbehl31@gmail.com

6 Biofunctional Molecule Exploratory (BMEX) Research Group, School of Pharmacy, Monash University Malaysia, Bandar Sunway 47500, Malaysia; goh.bey.hing@monash.edu

7 College of Pharmaceutical Sciences, Zhejiang University, Hangzhou 310058, China

8 Chemical Engineering Discipline, School of Engineering, Monash University, Selangor 47500, Malaysia; patrick.tang@monash.edu

* Correspondence: gokhanzengin@selcuk.edu.tr (G.Z.); stefano.dallacqua@unipd.it (S.D.); f.mahomoodally@uom.ac.mu (M.F.M.)

Preparation of extracts

Briefly, for the maceration (MAC), plant materials (5 g) were extracted with 100 ml of methanol or ethyl acetate for 24 h at a room temperature. In MAC, the extractions were performed with stir and not-stir. In the Soxhlet extraction procedure, plant materials (5 g) were extracted with 100 ml of methanol or ethyl acetate in a Soxhlet apparatus for 6 h. In the infusion extraction procedure, plant materials (5 g) were kept in boiled water (100 ml) for 15 min and afterwards filtered. Ethyl acetate and methanol extracts were concentrated using rotary-evaporator under vacuum. Infusions were dried using lyophilizator. All prepared extracts were stored in the dark at + 4 ˚C until further analysis.

Table S1. Eigen values and percentage of explained variance of each principal component (PC).

Principal component	PC1	PC2	PC3	PC4
Eigenvalues	6.91	2.29	1.94	1.07
Proportion of explained variance (%)	46.07	15.31	12.95	7.14
